# Developing key performance indicators for prescription medication systems

**DOI:** 10.1371/journal.pone.0210794

**Published:** 2019-01-15

**Authors:** Eldon Spackman, Fiona Clement, G. Michael Allan, Chaim M. Bell, Lise M. Bjerre, Dave F. Blackburn, Régis Blais, Glen Hazlewood, Scott Klarenbach, Lindsay E. Nicolle, Nav Persaud, Silvia Alessi-Severini, Mike Tierney, Harindra C. Wijeysundera, Braden Manns

**Affiliations:** 1 Department of Community Health Sciences and O’Brien Institute of Public Health, Calgary, Alberta, Canada; 2 Department of Family Medicine, University of Alberta, Edmonton, Alberta, Canada; 3 Department of Medicine, Sinai Health System and University of Toronto, Toronto, Ontario, Canada; 4 Department of Family Medicine, Bruyère Research Institute, and School of Epidemiology, Public Health and Preventive Medicine, University of Ottawa, Ottawa, Ontario, Canada; 5 College of Pharmacy and Nutrition, University of Saskatchewan, Saskatoon, Saskatchewan, Canada; 6 School of Public Health, Université de Montréal, Montreal, Quebec, Canada; 7 Department of Medicine, University of Calgary, Calgary, Alberta, Canada; 8 Department of Medicine University of Alberta, Edmonton, Alberta, Canada; 9 Rady Faculty of Health Sciences, University of Manitoba, Winnipeg, Manitoba, Canada; 10 St Michael's Hospital and University of Toronto, Toronto, Ontario, Canada; 11 College of Pharmacy and Manitoba Centre for Health Policy, Rady Faculty of Health Sciences, University of Manitoba, Winnipeg, Manitoba, Canada; 12 Independent Researcher, Ottawa, Onttario, Canada; 13 Canadian Agency for Drugs and Technology in Health (CADTH), Ottawa, Ontario, Canada; 14 Schulich Heart Center, Sunnybrook Health Sciences Center, University of Toronto, Toronto, Canada; RTI International Metals Inc, UNITED STATES

## Abstract

**Objective:**

To develop key performance indicators that evaluate the effectiveness of a prescription medication system.

**Methods:**

A modified RAND/UCLA appropriateness method was used to develop key performance indicators (KPIs) for a prescription medication system. A broad list of potential KPIs was compiled. A multidisciplinary group composed of 21 experts rated the potential KPIs. A face-to-face meeting was held following the first rating exercise to discuss each potential KPI individually. The expert panel undertook a final rating of KPIs. The final set of KPIs were those indicators where at least 80 percent of experts rated the indicator highly i.e. rating of ≥ 7 on a scale from 1 to 9.

**Results:**

292 KPIs were identified from the published literature. After removing duplicates and combining similar indicators 71 KPIs were included. The final ranking resulted in six indicators being ranked 7 or higher by 80% of the respondents and an additional seven indicators being ranked 7 or higher by ≥70 but ≤80% of respondents. The six selected indicators include four specific disease areas, measure structural and process aspects of health service delivery, and assessed three of the domains of healthcare quality: efficiency, effectiveness, and safety.

**Conclusions:**

These indicators are recommended as a starting point to assess the current performance of prescription medication systems. Consideration should be given to developing indicators in additional disease areas as well as indicators that measure the domains of timeliness and patient–centeredness. Future work should focus on the feasibility of measuring these indicators.

## Introduction

Effective prescription medication systems ensure patients can access needed medications, reduce overuse of inappropriate medications, and optimize use of cost-effective medications.[1,2] Prescription medication systems include, but extend beyond publicly funded drug plans. In many jurisdictions the prescription medication system includes physicians who prescribe medications, pharmacists that dispense and sometimes prescribe medication, the public and private prescription drug plans that provide partial or full payment for prescription medications and the patients who use prescription medications.

In a recent review of Canadian provincial drug plans many differences were noted in who was covered, what medications were covered, the total medication expenditures of each province, the government share of the total expenditure, and the out-of-pocket costs borne by the patient.[3] To inform decision making about the optimal organization of a prescription medication system it is important to have metrics to evaluate the effectiveness of a jurisdiction’s prescription medication system.

Key performance indicators (KPIs) are metrics that evaluate the outcomes of an organization, or of particular activities. The assessment of performance identifies gaps between current and desired outcomes and provides an indication of progress towards closing the gaps.[4] KPIs are commonly used to measure and improve upon the performance of healthcare systems; however, no KPIs have been specifically developed to evaluate or compare prescription drug systems across jurisdictions. The Canadian Institute for Health Information (CIHI) reports a broad collection of health indicators to help provinces/territories, regional health authorities and facilities monitor the health of their populations and track how well their health systems function.[5] In England, KPIs have been used through the Quality and Outcomes Framework (QOF) to standardize and incentivize improvements in the delivery of primary medical care.[6,7] While some of the QOF indicators encourage appropriate medication use, the focus of QOF is on primary health care delivery.

In this paper, we introduce a set of KPIs for measuring the performance of a prescription medication system and describe the process we used to develop them.

## Methods

We used a modified RAND/UCLA appropriateness method to develop KPIs for a prescription medication system.[8–10] This method is a formal group judgment process that combines collective judgment of experts with scientific evidence, by asking panelists to rate, discuss, and then re-rate indicators. It is used extensively to assess what constitutes appropriate and necessary care within a health care system.[11] In this modified procedure, four steps were taken: 1) Identifying published KPIs relevant to a prescription medication system; 2) Rating of KPIs by an expert panel; 3) Meeting of the expert panel to discuss and develop KPIs; and 4) A final rating of the modified list of KPIs by the expert panel. This study was approved by the ethics committee at the University of Calgary, REB16-1747.

### Identifying published KPIs

We compiled a broad list of potential KPIs. The following sources were searched: US Agency for Healthcare Research and Quality (AHRQ)[12]; National Health Services Quality Outcomes Framework (QOF)[13]; World Health Organization (WHO)[14]; Healthcare Effectiveness Data and Information Set (HEDIS)[15]; the Canadian Institute of Health Information (CIHI) Indicator Library[16]; The World Bank’s World Development and Service Delivery Indicators[17]; OECD’s Health Care Quality Indicators[18]; and Indicators of Quality Prescribing in Australian General Practice (a manual developed by Australia’s National Prescribing Service)[19]. We kept our initial search broad and included verbatim every KPI of relevance to a prescription drug system from the published source. Similar indicators were combined to represent a single indicator and those that were not relevant to the Canadian system were excluded, for example ‘The estimated number of children eligible for antiretroviral therapy’ (because the prevalence of HIV is so low in children in Canada this would not have been a useful marker of appropriate medication access).

### Rating of KPIs by an expert panel

A multidisciplinary group composed of 21 experts in drug evaluation, clinical therapeutics, drug policy, drug plan management, and/or performance indicator development were invited by email and consented to participate. Each expert was sent the list of identified published KPIs and a brief description of its intent and purpose. Experts were instructed to identify KPIs that satisfied three criteria: a) likely associated with patient outcomes; b) could be influenced by drug plan policies; and c) higher performance on the indicator would be considered a higher quality prescription drug system.

The experts were asked to assess the validity of each proposed indicator on two scales: 1) importance to quality care and health of the patient; and 2) sensitivity to performance of drug coverage i.e. how well the indicator is likely to change given changes in drug plan policies. Scales ranged from 1 (Not valid) to 9 (Extremely valid). Experts were also asked if they had any suggestions or comments.

### Meeting of the expert panel to discuss and develop KPIs

A face-to-face meeting was held following the first rating exercise to discuss each quality indicator individually. Experts were provided with a summary of the first-round ratings and a confidential reminder of their own previous rating. The experts were divided into small groups and asked to discuss in detail a group of indicators which shared similar content areas. The discussion focused on the evidence (or lack thereof) supporting or refuting each indicator and the first-round ratings of the experts. Experts were also given the opportunity to propose alternate wording for each indicator or propose additional indicators. They were then asked to separate indicators into bins marked “Very good”, “Good”, or “Poor”. The small groups were then re-organized and the new groups reviewed the first group’s judgments. After the results of the second small groups were collated and separated into “Very good”, “Good”, or “Poor”, the entire panel met as a group. During the final session of the day, indicators that were noted to be poor were reviewed and removed, if consensus could be achieved. Comments on specific KPIs were also discussed, and instructions were provided with respect to the second round of confidential rating. During this discussion expert members noted that additional indicators relevant to step therapy should be developed. Step therapy is an approach to prescription intended to control the costs and risks by only progressing to more costly or risky therapies within specific clinical areas if necessary.

### Final rating of modified list of KPIs by the expert panel

After creation of the additional KPIs as noted above, all remaining indicators were re-rated. Respondents were asked to consider whether each metric was appropriate as a performance indicator of a prescription medication system, using a scale from 0 (Not appropriate) to 9 (Completely appropriate). Consistent with the RAND methodology, the final set of indicators included those indicators where at least 80 percent of experts rated the appropriateness of the indicator highly (rating of ≥ 7).[10] Results were discussed with the experts in a series of conference calls to inform them of the results and understand their perception of the results.

## Results

### Identifying published KPIs

A total of 292 KPIs were identified from the published literature. After removing duplicates and combining similar indicators, a total of 108 KPIs were included. After initial discussions with the research team with the goal of excluding KPIs of very low relevance, the list of 108 indicators was reduced to 71 indicators for review by the expert panel.

### Rating of KPIs by an expert panel

The first rating exercise was completed by 18 of 21 (86 percent) of the experts. The results of the first rating exercise were summarized and returned to each respondent. An example of the personalized information provided to each of the respondents for each of the 71 indicators is provided in S1 Table.

### Meeting of the expert panel to discuss and develop KPIs

Of the 21 experts who were invited to the face-to-face meeting, two were unable to attend. A number of important decisions and actions arose from this meeting, including revisions to the wording of many indicators, the need to represent overall prescription drug systems rather than drug insurance activities only, and the need to develop some additional indicators for step therapy. It was also decided at the meeting to identify indicators by the Donabedian outcome framework (structure, process and outcome)[20] and the Agency for Healthcare Research and Quality (AHRQ) outcome framework (effective, safe, efficient, equitable, timely, and patient-centered)[21].

With respect to the need to focus on the entire prescription medication system, it was noted that poor performance across KPIs could be attributable to many stakeholders, including the public drug plan, private insurance plans, physicians, and pharmacists. The experts decided that it may be difficult to disentangle the effects of each of these actors on KPIs. Rather than try to develop KPIs that would solely be affected by the prescription drug plan, it was decided that a broader view would be appropriate and that indicators could represent any aspect of the prescription drug system.

The group also discussed whether the ratings of the public drug plan managers included in the expert panel should be used in the final ranking as a perception of bias could be raised given that the indicators developed may be used to assess the performance of the drug plans. After discussion, it was decided that the drug plan manager’s responses would be included because of their expertise in the drug system, but a sensitivity analysis, with and without the responses of the drug plan managers, was undertaken.

Through group discussion, 43 indicators were considered poor and were excluded from further rating; 28 of the 71 indicators were considered appropriate for further rating. After completion of the in-person meeting, the experts considered it important to include indicators of adherence to step therapy. Six additional step therapy indicators were developed with the help of three clinical experts. These indicators focused on the treatment of diabetes, chronic obstructive pulmonary disease (COPD), and rheumatoid arthritis. These diseases were selected because they are common, because of the strong evidence base regarding step therapy and the existence of national treatment guidelines.

### Final rating of published and newly developed KPIs by the expert panel

The second and final rating exercise asked respondents to rate 34 KPIs. After the meeting each author was sent an electronic survey to complete. Of the 21 experts invited to undertake the final rating exercise 17 (81 percent) completed the survey. The respondents had broad expertise and were from a variety of provinces (Table 1).

**Table 1:** Demographic characteristics of the second survey respondents.

Category	Demographic Characteristics	No. of respondents	Percentage of total (%)
**Gender**	Male	12	71
	Female	5	29
**Experience**	< 5 years	5	29
	5 to 10 years	3	18
	10 to 15 years	4	24
	15 to 20 years	4	24
	> 20 years	1	6
**Current** **Title**	Academic physician	5	29
	Non-academic physician	3	18
	Academic	5	29
	Drug plan manager	3	18
	Health care administrator	1	6
**Areas of Expertise**	Drug policy	12	71
	Health economics	10	59
	Measurement	3	18
	Cardiology	1	6
	Infectious disease	1	6
	Intensive care	1	6
	Nephrology	2	12
	Public health	1	6
	Other	5	29
**Province**	Alberta	6	35
	BC	1	6
	MB	2	12
	NB	1	6
	ON	6	35
	SK	1	6

Six indicators were ranked 7 or higher by 80% of the respondents (Table 2),

1. The proportion of patients taking a brand name medication where a therapeutically equivalent lowest cost generic is available in class.
2. The price in each province for a basket of selected medicines.
3. In adults (age ≥ 30), initiating pharmacologic management of type 2 diabetes, the proportion who receive metformin as first-line therapy.
4. The proportion of patients with asthma (age < 45) prescribed a long acting beta2 agonist who are NOT using an inhaled corticosteroid.
5. In adults (age > 18) with rheumatoid arthritis who are receiving biologic or targeted synthetic therapy, the proportion of patients who had previously received methotrexate and at least one other traditional DMARD, either alone or in combination.
6. The proportion of patients (age ≥ 18) diagnosed with chronic non-cancer pain dispensed an opioid at a dose less than the equivalent of 100mg per day of morphine.

**Table 2:** The six final key performance indicators determined to be valid.

Key Performance Indicator	Disease	Rationale	Dimensions of Care	Domains of Healthcare Quality	*Poor performance could be due to issues with the following stakeholder groups within the medication care system*		
			*Structure*; *Process*; *Outcome*	*Safety*; *Effectiveness*; *Patient-centeredness*; *Timeliness*; *Efficiency*; Equity*			
					Drug Plan	Physician	Pharmacist
1) The proportion of patients taking a brand name medication where a therapeutically equivalent lowest cost generic is available in class.	All	Since generic medications are almost always bioequivalent but less expensive, using a higher proportion of brand name medications (where generics are available) would be an inefficient use of resources.	Structure; Process	Efficiency	Yes	Yes	Yes
2) The price in each province for a basket of selected medicines.	All	Higher prices for medicines in one province would highlight areas where price negotiation should be pursued.	Structure	Efficiency	Yes	—	—
3) In adults (age ≥ 30), initiating pharmacologic management of type 2 diabetes, the proportion who receive metformin as first-line therapy.*	Diabetes	Guidelines recommend metformin as first-line therapy, as it has been shown to improve outcomes and it is inexpensive. Since it is contraindicated in some patients, we would not expect 100 percent compliance with this, but provinces where more patients are starting antidiabetic therapy with non-metformin agents should explore this variation with the goal of maximizing first-line use of metformin.	Process	Effectiveness; Efficiency; Safety	Yes	Yes	Yes
4) The proportion of patients with asthma (age < 45) prescribed a long acting beta2 agonist who are NOT using an inhaled corticosteroid.*	Asthma	Guidelines recommend use of inhaled corticosteroids as first line therapy in asthma (more common in those aged < 45), and that long acting beta2 agonist should be add-on therapy in those with an insufficient response to inhaled corticosteroid.	Process	Effectiveness; Efficiency; Safety	Yes	Yes	Yes
5) In adults (age > 18) with rheumatoid arthritis who are receiving biologic or targeted synthetic therapy, the proportion of patients who had previously received methotrexate and at least one other traditional DMARD, either alone or in combination.*	Rheumatoid arthritis	All provincial publicly funded drug formularies mandate use of DMARDS prior to use of biologic therapy in rheumatoid arthritis, since first line use of biologic agents in rheumatoid arthritis is not cost-effective.	Process	Efficiency	Yes	Yes	Yes
6)The proportion of patients (age ≥ 18) diagnosed with chronic non-cancer pain dispensed an opioid at a dose less than the equivalent of 100mg per day of morphine.*	Chronic pain	There are many concerns with increasing opioid use in society. Guidelines discourage the use of opioids in patients without cancer for the management of chronic pain. Patients on less than 100 mg of morphine equivalent should not be on opioids at all.	Process	Safety; Effectiveness	Yes	Yes	Yes

*subgroup analysis within each KPI would inform equity consideration

In the sensitivity analysis, removing the drug plan manager’s responses resulted in no changes to which indicators were ranked 7 or higher by ≥80% of respondents.

An additional seven indicators were ranked 7 or higher by ≥70% but ≤80% of respondents (S2 Table).

## Discussion

A panel of 21 experts considered a list of 71 KPIs of a prescription medication system, and selected six indicators as being valid KPIs. The selected indicators include four specific disease areas, measure structural and process aspects of health service delivery, and assessed three of the domains of healthcare quality: efficiency, effectiveness, and safety. None of the included indicators measured health outcomes or reflected timeliness or patient–centeredness. While none of the included indicators specifically measured equity, KPI’s 3–6 in Table 2, when measured across a variety of patient subgroups would enable an assessment of equity.

While the initial goal of this project was to develop indicators to measure the performance of prescription drug plans, discussion of the experts at the face-to-face meeting recommended it was more appropriate to develop KPIs reflecting performance of the overall prescription medication system. All agreed that it would be impossible to disentangle the effect of the many different stakeholders that contribute to the health of patients through the use of prescription medications (including publicly funded drug plans, private drug plans, physicians and pharmacists). However, experts involved felt that the selected KPIs would enable the comparison of prescription medication system performance across jurisdictions, acknowledging that performance variations could be due to a variety of factors within a system. Experts noted that understanding differences in jurisdictional performance on a particular indicator would require consideration of the jurisdictions’ demographics, the provincial health care system and the capacity of provincial drug plans. When using these KPIs, experts recommended detailed interviews with the drug plan managers to understand their current rules and regulations, and their ability to optimize appropriate use of medications within their drug plan.

The experts agreed that these indicators are not meant to judge the performance of a publicly funded drug plan, nor its managers, as each jurisdiction has evolved differently and faces different constraints (including varied budget and legal responsibilities). Moreover, as noted, many aspects of the prescription medication system will affect these metrics.

The ability to develop indicators is limited by the available evidence on optimal prescribing in disease areas. There was some concern that measuring indicators in only four disease areas would put undue attention on these diseases. This might result in prescription medication systems focusing improvements in these narrow disease areas in order to improve outcomes on these KPIs. Experts were careful to note that these should be seen as representative disease areas–and that lower or higher performance in these indicators would likely extend to other disease areas.

It was recommended that the next steps would be to develop technical definitions and measure the selected KPIs using administrative health data (where needed) across different jurisdictions to assess feasibility and establish baseline performance. An additional seven indicators (Appendix 2) were also recommended for measurement and further evaluation, based on having been ranked as a valid indicator by at least 70% of the expert group. Experts noted the importance of future meetings to consider the results of the initial evaluation, and to develop additional KPIs that reflect the performance of a prescription medication system, and possibly to remove existing KPIs as best practices change.

The prescription medication system is an important part of overall health care and plays an important role in maximizing the health of the population through cost-effective provision of medications. There are many differences in prescription medication systems across jurisdictions: eligibility criteria for beneficiaries, types of medicines covered, patient copayments, and total expenditures. KPIs were developed to evaluate the effectiveness of different prescription medication systems and inform decision makers about changes in organization.

## Conclusion

A multifaceted process involving a panel of 21 experts developed and assessed KPIs of a prescription medication system. Multiple rounds of rating, conference calls and one face-to-face meeting reduced the initial list of 292 indicators to six indicators rated highly as being valid KPIs. These indicators are recommended as a starting point to assess the current performance of prescription medication systems across different jurisdictions. Consideration should be given to developing indicators in additional disease areas as well as indicators that measure the domains of timeliness and patient–centeredness. In order to understand differences–and improve performance within KPIs across jurisdictions—assessing the capacity of publicly funded medication systems will be important. Future work will focus on the feasibility of measuring these indicators.

## Supporting information

S1 Table Example response from the first rating exercise. (DOCX)

S2 Table The seven key performance indicators that were highly ranked by 70 percent of respondents. (DOCX)
