# Supplementary material for: Developing key performance indicators for prescription medication systems
Source: PLoS One. 2019 Jan 15;14(1):e0210794. doi: 10.1371/journal.pone.0210794 (PMC6333341; doi:10.1371/journal.pone.0210794)
Supplement: S1 Table — (DOCX) [file pone.0210794.s001.docx]

Supporting Information 1: Example response from the first rating exercise.

| **The percentage of patients taking a generic where a brand is available in class.**    *Importance to Quality Care and Health of the Patient*  1 2 3 4 5 6 7 8 9 UA  ***Frequency 4 1 1 1 2 3 5 0 1 0***  *  *Sensitivity to Performance of Drug Coverage*  1 2 3 4 5 6 7 8 9 UA  ***Frequency 0 0 0 1 1 3 6 2 5 0 ✓***  * |
| --- |

0 = no importance/sensitivity, 9 = extreme importance/sensitivity

In this example, respondents generally considered the indicator to be less important for quality care and the health of the patient; the respondent receiving this report had rated this indicator a 6 on this scale. The sensitivity of the indicator to the performance of the prescription drug system was considered higher; this respondent rated this indicator 9 on this scale. The check mark signifies that more than 80 percent of respondents rated this indicator 7 or higher on this scale. Respondents reported a number of comments, which were made available to all participants.
