# Supplementary material for: Developing key performance indicators for prescription medication systems
Source: PLoS One. 2019 Jan 15;14(1):e0210794. doi: 10.1371/journal.pone.0210794 (PMC6333341; doi:10.1371/journal.pone.0210794)
Supplement: S2 Table — (DOCX) [file pone.0210794.s002.docx]

Supporting Information 2: The seven key performance indicators that were highly ranked by 70 percent of respondents

| **Key Performance Indicator** | **Disease** | **Dimensions of Care** | **Domains of**  **Healthcare Quality** | ***Poor performance could be due to issues with the following stakeholder groups within the medication care system*** | | |
| --- | --- | --- | --- | --- | --- | --- |
|  |  | *Structure*;  *Process*;  *Outcome* | *Safety*; *Effectiveness*; *Patient-centeredness*; *Timeliness*; *Efficiency*; *Equity** |  |  |  |
|  |  |  |  | **Drug Plan** | **Physician** | **Pharmacist** |
| 1. The proportion of patients who did not refill a prescription for a chronic medication.* | All | Structure;  Process | Effectiveness | Yes | Yes | Yes |
| 1. The proportion of patients at high cardiovascular risk (e.g., diabetes over age 40; or those with known cardiovascular disease or those with 10-year coronary heart disease risk >10%) who were dispensed a statin and of those, the proportion who are using statins for at least 80% of a one-year period.* | Cardio-vascular | Structure;  Process | Effectiveness;  Patient-centeredness | Yes | Yes | Yes |
| 1. The proportion of patients discharged after a myocardial infarction who were prescribed and dispensed ACE-I or ARB, aspirin or an alternative anti-platelet therapy, and statin in the next year, and of those, the proportion who are using all for at least 80% of a one-year period.* | Myocardial Infarction | Structure;  Process | Effectiveness;  Patient-centeredness | Yes | Yes | Yes |
| 1. The proportion of patients (age ≥ 18) with both an ischemic stroke and atrial fibrillation / flutter who are prescribed and dispensed anticoagulation therapy (warfarin or any direct oral anti-coagulant [DOAC]) at hospital discharge, and the proportion receiving anticoagulation that receive a DOAC.* | Stroke | Process | Effectiveness;  Patient-centeredness;  Efficiency | Yes | Yes | Yes |
| 1. The proportion of patients with a non-specific upper respiratory tract infection (URTI) dispensed an antibiotic.* | Infection | Process | Efficiency;  Safety | Yes | Yes | Yes |
| 1. The percentage of patients (age > 65) dispensed benzodiazepines or one of the Z-drugs (including zopiclone, zolpidem and zaleplon) for more than 4 weeks.* | Insomnia | Process | Safety | Yes | Yes | Yes |
| 1. For adults (age > 40) on inhaled therapies for COPD, the proportion of patients receiving third-line therapy (LAMA/LABA or ICS/LAMA).* | COPD | Process | Efficiency | Yes | Yes | Yes |

*subgroup analysis within each KPI would inform equity considerations
